# Low Cooking Skills Are Associated with Overweight and Obesity in Undergraduates

**DOI:** 10.3390/nu15112424

**Published:** 2023-05-23

**Authors:** Rafaela Nayara da Costa Pelonha, Manuela Mika Jomori, Tamara Gonçalves Maciel, Jéssica Adla Dantas Rocha, Thaís Souza Passos, Bruna Leal Lima Maciel

**Affiliations:** 1 Graduate Program in Health Sciences, Federal University of Rio Grande do Norte, Natal 59078-970, Brazil; rafaelanayara52@gmail.com (R.N.d.C.P.); thais.passos@ufrn.br (T.S.P.); 2 Department of Nutrition, Federal University of Santa Caratarina, Florianopolis 88040-900, Brazil; manuela.jomori@ufsc.br (M.M.J.); tamaramaciel21@hotmail.com (T.G.M.); 3 Department of Nutrition, Federal University of Rio Grande do Norte, Natal 59078-970, Brazil; jessicaadla2503@gmail.com

**Keywords:** culinary skills, COVID-19, undergraduate students

## Abstract

Culinary skills are defined as the confidence, attitude, and the application of one’s individual knowledge in performing culinary tasks, and their development may be associated with better diet quality and better health status. This study aimed to analyze the association between cooking skills, overweight, and obesity in undergraduates. This is a descriptive, observational, and cross-sectional study, with data collected between October 2020 and March 2021, with undergraduate students (*n* = 823) at the Federal University of Rio Grande do Norte. Participants answered the online Brazilian Cooking Skills and Healthy Eating Questionnaire Evaluation, BCSQ, which included socioeconomic information. Logistic regressions were used to assess the associations of cooking skills with overweight and obesity. From the total of the students, 70.8% were female, with a median age of 23 (21–30) years; 43.6% were with overweight or obesity; 48.8% were eutrophic; and 7.7% underweight. Overweight and obesity were significantly associated with low levels of culinary self-efficacy and self-efficacy in the use of fruits, vegetables, and seasonings in the bivariate analysis. The logistic regressions showed that living with other people and eating out were associated with higher chances of overweight and obesity. Sharing the responsibility for preparing meals and a high self-efficacy in the use of fruits, vegetables, and seasonings were associated with lower chances for overweight/obesity. Overall, our study showed that overweight and obesity were associated with lower cooking skills in the studied undergraduates. Therefore, the study demonstrates that culinary skills can be explored in educational programs that aim to reduce overweight/obesity in students.

## 1. Introduction

Cooking skills are defined as the confidence, attitude, and the application of one’s individual knowledge in performing culinary tasks, such as planning meals, shopping, and preparing different kinds of food (fresh and/or processed ones) [1]. Studies have shown the importance of the development of cooking skills for adopting healthy eating habits because homemade foods favor the consumption of vegetables, improving the nutritional quality of the meals [2,3,4,5].

Undergraduate students have a high consumption of ultra-processed foods. Fondevila-Gascón [6], for example, observed that 83% of their university student sample consumed snacks and nuggets weekly, as well as soft drinks and pastries. According to Monteiro et al. [7] ultra-processed foods are multi-ingredient formulations with food substances that mimic unprocessed foods’ sensory qualities. The high consumption of this type of food, which requires little or no preparation technique, is related to the sporadic use of cooking skills [4,8].

The increased consumption of this type of food among university students can be explained by the fact that some young adults face an unhealthy lifestyle triggered by the transition from adolescence to adulthood, a period characterized by the search for autonomy and increased responsibilities [9,10]. In this time of life, having little time for cooking is typical. Some of the reasons for limited cooking time include the easy access to convenience foods and the academic routine, which imposes the need to balance the time between university activities, studies, work, and social life [9,11]. In addition, students who live in campus residences or with their families usually have their meals prepared by others, which facilitates the belief that having a healthy diet is a burden [12]. Beyond this, students in residence would require access to domestic kitchens in order to cook more as communal kitchens may not present adequate conditions for preparing meals [13].

In this context, university students can develop overweight and, consequently, obesity. Obesity is a chronic disease recurrently associated with severe complications, which can cause or worsen other diseases and, consequently, reduce the life expectancy of affected individuals. The etiology of obesity is quite complex and includes economic, cultural, political, and individual factors [14,15]. At an individual level, obesity can be associated with lifestyle and result from reduced physical activity combined with dysfunctional eating habits and consuming energy-dense foods, contributing to weight gain from excessive calorie intake [15]. Given this, dysfunctional eating habits are consequences of the nutritional transition we are experiencing in the contemporary world. This transition involves shifting from fresh to ultra-processed foods and changing how people obtain and prepare food [7,16].

With the pandemic’s beginning, universities closed their facilities and adhered to remote learning, resulting in students facing an unexpected and potentially anxiety-generating situation [17,18]. Therefore, the pandemic scenario negatively influenced eating habits and, specifically, increased food intake as people’s anxieties triggered them to use food to respond to negative emotions [19,20]. Consequently, the increase in food intake associated with the greater consumption of processed foods may be related to the emergence of overweight and obesity in undergraduate students [20].

It is important to emphasize that, in Brazil, undergraduate students present a particularly social vulnerability because the profusion of student assistance programs exclusively for the public, such as ProUni, FIES, and PNAES [21,22,23], has increased over recent decades. In addition, many of these students are served by university restaurants, which had to cease their operation due to the pandemic, increasing vulnerability by compromising even more the food security of these students.

In addition, the pandemic brought some protective measures to prevent its spread, such as social distancing and lockdown [24]. This generated changes in people’s food consumption, such as reduced trips to the supermarket and an increase in the use of delivery services [25]. As a result, the intake of fruits and vegetables reduced, and the consumption of ultra-processed foods increased [26]. On the other hand, the COVID-19 pandemic also contributed to the increase in the preparation of homemade food; however, university students also increased the use of ultra-processed foods as part of their preparations [27].

Considering lifestyle changes from adolescence to adulthood, which impact lifestyle and, thus, food preparation and eating, and the scenario of the COVID-19 pandemic, which also changed eating habits, the present study aimed to observe the association between cooking skills, overweight, and obesity in undergraduates. The hypothesis under study is that undergraduates with lower levels of cooking skills present higher chances of being with overweight or obesity.

## 2. Materials and Methods

### 2.1. Ethics

This study is part of a larger, multicenter project entitled: “Nutrition is in the kitchen! Cooking skills and healthy eating at the university”, which takes place at the Federal University of Santa Catarina—UFSC (coordinating center), the Federal University of Rio Grande do Sul—UFRGS, the Federal University of Alagoas—UFAL, and the Federal University of Rio Grande do Norte—UFRN, with an international partnership with the City University of London. The local project was approved by the Ethics Committee of the Onofre Lopes University Hospital (CAAE 36572420.1.0000.5292, number 4.523.788). All volunteers registered their online consent to participate in the study.

### 2.2. Study Design and Participants

This is a cross-sectional study, with data collection between October 2020 and March 2021. The research was directed to all regularly enrolled undergraduate students from the Federal University of Rio Grande do Norte, and the inclusion criterion was to be regularly enrolled in an undergraduate course at UFRN. The research was directed to all students of the university campus; that is, it included both students who received assistance to live in the university residence, as well as those who lived in their own homes. However, during the research, the university residence remained closed, so the students had to return to their parents’ homes [28].

The sample size was determined by considering 29,291 undergraduate students at UFRN in 2019; a prevalence of overweight of 40%; an error of 5%; a design effect of 2.0; and losses of 10%, totaling 809 students. The invitation to participate in the research and the informed consent form were sent to institutional electronic addresses (e-mails). The study questionnaire was answered online, using the Google Forms platform (Google Forms). Study participants were selected after volunteering, without randomization in the selection, and totaled 846 responses. Twenty-four adolescents were excluded from the present study. Thus, the total number of participants was 822.

### 2.3. Sociodemographic Characterization, Meal Preparation, and Consumption

The questionnaire had 15 questions about sociodemographic variables, meal preparation, and consumption characteristics. The questions sought information about gender, date of birth, undergraduate courses, when the participant was enrolled in university, and whether he/she was a beneficiary of any local university aid program. They also covered parental education, ethnicity, civil status, and whether they had children. Regarding the characteristics of meal preparation, there were questions about the time available to cook per day, the people responsible for preparing food at home, the equipment and kitchen utensils available for cooking, and where the main meal was consumed [29].

### 2.4. Nutritional Status

Weight (Kg) and height (m) data were self-reported by the participants in the questionnaire, and the values were used to calculate the body mass index (BMI). After calculation, the BMI was classified according to the World Health Organization [30].

### 2.5. Cooking Skills

The Brazilian questionnaire for the assessment of cooking skills and healthy eating (BCSQ) was used. The questionnaire is the result of an adaptation of the Cooking with Chef Program and was validated for Brazil (Cronbach’s alpha = 0.70) [31,32,33]. The internal consistency of the BCSQ was also calculated in our sample, and the Cronbach’s alpha was 0.70.

The BCSQ presents 36 questions distributed on 7 scales: (1) availability of fruits and vegetables (8 items); (2) culinary attitude (4 items); (3) culinary behavior (3 items); (4) culinary self-efficacy (6 items); (5) self-efficacy in the consumption of fruits and vegetables (3 items); (6) self-efficacy in the use of fruits, vegetables, and seasonings (4 items); (7) knowledge of culinary terms and techniques (8 items).

Scales 2–6 were considered for calculating the level of cooking skills, and these were classified as low (20–43 points), medium (44–73 points), or high (74–100 points). Availability of fruits and vegetables was classified as low (0–2 points), medium (3–6 points), or high (7–8 points). The knowledge of culinary terms and techniques was classified as high when the participant correctly answered ≥6 items or low when the participant answered ≤ 5 items correctly [29,31].

### 2.6. Statistical Analysis

The data obtained were moved from Google Forms to the Microsoft Excel program (2013) and coded for the analysis performed using the Statistical Package for the Social Sciences SPSS^®^, version 18.0 (IBM Corporation, Armonk, NY, USA, 2011).

For the students’ characterization variables, a descriptive analysis of categorical variables was performed by the distribution of the absolute (n) and relative (%) frequency and the discrete and continuous variables by the median (Q1–Q3), according to the non-normality of the data, and verified using the Kolmogorov–Smirnov test.

Association tests were performed between the results of the BCSQ, the socioeconomic and meal preparation variables, and the presence of overweight or obesity. The chi-square test and Fisher’s exact test were used, with the latter being used for the 2 × 2 tables.

The variables that showed a significant association in the univariate analysis with the presence of overweight or obesity were used for logistic regression models, first exploring the effect of a single variable in the presence of overweight or obesity (1 = yes, 0 = no), and their unadjusted odds ratios (OR) and respective 95% confidence intervals (95% CI) were demonstrated. Then, adjusted logistic regression models were calculated, considering the presence of overweight or obesity (1 = yes, 0 = no) as the dependent variable. The adjustment of the final model shown was guaranteed by observing the Omnibus test, with *p* values less than 0.05, and the Hosmer and Lemeshow test, considering *p* values greater than 0.05. Thus, sex, income, living arrangement, responsibility for preparing meals at home, place where the main meal was consumed, and scales of cooking skills (culinary self-efficacy and self-efficacy in the use of fruits, vegetables, and seasonings) were included in the final model as independent variables. Multicollinearity between the independent variables was tested and tolerance and VIF values in the final adjusted model were approximately 1.0. The adjusted odds ratios (AOR) and their respective 95% CI were presented.

## 3. Results

### 3.1. Population Characterization

Undergraduates were, on average, 23 (21–30) years old, with a predominance of female students (70.8%), who declared themselves to be white (52.1%) and brown (37.7%) and who lived with either their parents or grandparents (61.3%). Twenty-nine percent of the studied undergraduates presented overweight, while 14.6% presented obesity. In addition, most presented an income >1.5 times the minimum wage (87.1%), some received student benefits (31%), and some had children under 16 years of age (12%) (Table 1).

Considering gender, 86.9% of the women declared knowing how to cook, 51.5% declared having ≥2 h a day available for cooking, and 41.6% were with overweight or obesity. On the other hand, 78.8% of the men declared knowing how to cook, 62.1% had ≥2 h a day available for cooking, and 48.3% were with overweight or obesity (Table 2).

### 3.2. Cooking Skills

**Figure 1:**
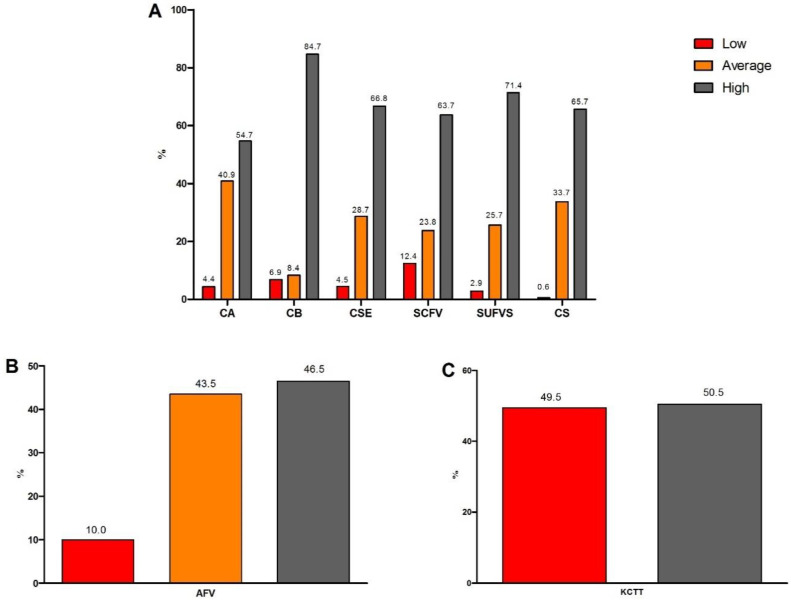
Cooking skills of the studied university students (*n* = 822), according to the Brazilian questionnaire for the assessment of cooking skills and healthy eating (BCSQ). (**A**) University students according to the levels in the BCSQ scales used to evaluate overall cooking skills, considering the sum of the scales: culinary attitude (CA); culinary behavior (CB); culinary self-efficacy (CSE); self-efficacy in the consumption of fruits and vegetables (SCFV); self-efficacy in the use of fruits, vegetables, and seasonings (SUFVS); and overall cooking skills (CS). (**B**) University students according to the availability of fruits and vegetables (AFV). (**C**) University students according to the knowledge of culinary terms and techniques (KCTT).

Most of the students (65.7%) presented high cooking skills; 33.7% had medium cooking skills; and 0.6% had low cooking skills. Most of the students presented high levels of culinary attitude (54.7%), culinary behavior (84.7%), culinary self-efficacy (66.8%), self-efficacy in the consumption of fruits and vegetables (63.7%), and self-efficacy in the use of fruits, vegetables, and seasonings (71.4%) (Figure 1A). Almost half of the students (46.5%) had a high availability of fruits and vegetables (Figure 1B), and half of the students (50.5%) showed a high knowledge of culinary terms and techniques (Figure 1C).

### 3.3. Association of Meal Preparation and Consumption with Overweight and Obesity

There were more students with overweight and obesity who ate meals out of the home than those without overweight or obesity (14.8% vs. 8.4%, respectively; Table 3). There were also more students with overweight and obesity who were solely responsible for preparing meals than those without overweight or obesity (26.5% vs. 17.2%, respectively; Table 3). For those who received help from someone else in preparing meals, there were more students without overweight or obesity who received help from their father than those with overweight or obesity (14.8% vs. 9.1%, respectively; Table 3). Considering the living arrangement, more students with overweight or obesity lived with a partner/spouse (13.1% vs. 7.3%, Table 3) or with a partner/spouse and children (14.5% vs. 5.4%, Table 3).

### 3.4. Association of Cooking Skills with Overweight or Obesity

There was a higher percentage of students with overweight or obesity presenting low levels of cooking self-efficacy (Chi-square, *p* = 0.026, Table 4) and low self-efficacy in the use of fruits, vegetables, and seasonings (Chi-square, *p* = 0.042, Table 4) compared with the students without overweight or obesity (5.9% vs. 3.4%, and 4.5% vs. 1.7%, respectively).

### 3.5. Logistic Regression for Variables Associated with Overweight and Obesity in the Studied Undergraduates

The sociodemographic variables and cooking skill scales associated with overweight and obesity were tested using logistic regressions (Table 5). The logistic regression showed that living with a partner/spouse (AOR = 3.17; 95%CI = 1.42–7.04), living with a partner/spouse and children (AOR = 5.10; 95%CI = 2.17–12.00), and consuming the main meal out of the home (AOR = 1.89; 95%CI = 1.14–3.13) increased the chances of overweight and obesity.

On the other hand, some variables reduced the chances of overweight or obesity: sharing the responsibility for preparing meals with one person (AOR = 0.44; 95% CI = 0.26–0.74); having another person responsible for preparing the meals (AOR = 0.42; 95%CI = 0.22–0.80); and a high self-efficacy in the use of fruits, vegetables, and seasonings (AOR = 0.32; 95%CI = 0.11–0.95).

## 4. Discussion

This study analyzed the association between cooking skills, overweight, and obesity in university students. Our data showed that living with a partner/spouse and children and eating the main meal out of the home were associated with overweight and obesity. However, sharing responsibility for preparing meals was associated with a lower chance of being with overweight or obesity. In addition, high levels of self-efficacy in the use of fruits, vegetables, and seasonings also reduced the chances of overweight and obesity.

We found that living with a partner/spouse and children increased the chances of overweight and obesity. Consistently with this finding, Lancaster [34] found an association between living with a spouse or partner and overweight/obesity in both men and women but with a stronger association for women. Another study [35] showed that the never-married population had the lowest prevalence of overweight and obesity when compared to those married, divorced, and widowed. Thus, the association of the marital status with overweight/obesity can be explained mainly by the increase in moments that encourage the regular consumption of meals, in addition to larger portions, resulting in greater energy intake, as well as a decrease in physical activity and a decrease in the desire to maintain bodyweight to attract a partner [36]. From these perspectives and considering the pandemic scenario, students were overwhelmed by distance learning. Still, they had to dedicate themselves to domestic activities, in addition to having to dedicate themselves to children who were in distance education [37]. These facts might explain why undergraduates living with a partner/spouse and children presented more chances for overweight and obesity in the present study.

However, on the other hand, Davis et al. [38] found that women who lived with their spouses had a lower BMI than those who lived with other people. Van Der Horst et al. [3] found that students who lived alone had a higher consumption of ready-to-eat meals compared to students who lived with other people, and this consumption of ready-to-eat meals was significantly associated with overweight and obesity. Based on these conflicting results, further studies are needed to understand better the association between housing conditions, meal consumption, and nutritional status, which may be also associated with socioeconomic variables.

Another factor associated with overweight and obesity in the present study was the responsibility of preparing meals. The logistic regression showed that sharing this responsibility with one more person or having another person cook the meal was associated with a lower chance of overweight and obesity. The Food Guide for the Brazilian Population highlights the importance of sharing part or all the activities that precede and follow the consumption of meals, including planning, acquisition, and preparation [5]. The responsibility of cooking for the family generally falls on the women, reinforcing the gender roles. Therefore, when cooking is divided among family members, the health status of the people involved is improved [5,39].

Additionally, we found in the bivariate analysis that more students with overweight or obesity reported being solely responsible for preparing their meals compared to those without overweight or obesity. Few studies have addressed the relationship between overweight and obesity and its association with the responsibility to cook at home. Ducrot et al. [40] evaluated the involvement in the choice of dishes for the home meal preparation of over 50 thousand French adults by an ongoing web-based prospective observational cohort study launched in 2009 with a scheduled follow-up of 10 years. The authors found that participants with overweight and obesity were more likely to choose dishes for home meals that were part of diets to lose weight. Additionally, their results suggested that participants with overweight and obesity were less likely to consider the importance of time for cooking, the availability of ingredients, and leftovers at home for planning meals [40].

Thus, in the context of undergraduates, having someone to help with meal preparations or another person to prepare the meals might have decreased the burden of house activities, stress, and help to maintain nutritional status. These facts might explain our results, and further studies should investigate living arrangements that help undergraduates in Brazil have a better quality of life.

In our study, more women reported knowing how to cook and having more time available for cooking than men. Women also presented higher levels of cooking skills and less overweight and obesity. These data are consistent with the results observed by Murakami et al. [41], suggesting that the difference in competence between genders is because women still have the greater responsibility for cooking and buying food. Our data are also consistent with the study by Dezanetti et al. [27], also studying undergraduates and using the BCSQ, which found that in the period before and during the pandemic, women were more likely to cook “several times a week”. Over the years, there has been a tendency for an increase in the time men dedicate to cooking (from 35% to 46% in 13 years) [42]. However, women still dedicate more time to cook than men. This fact could have several explanations. For example, men may cook more for pleasure and entertainment, while women, in the majority, cook as a family routine, which strengthens social norms around gender and makes women more involved in cooking, feel more confident, and pass on these skills to their children. Wolfson et al. [43] and Taillie [42] found that women cooked more frequently than men [42,43], like our findings. Therefore, the present study shows that meal preparation remains a highly gender-related task and that sharing this task with other family members contributes positively to health.

Conversely, we observed that men had lower levels of cooking skills than women. This result was also observed in other studies, such as in the research by García-González et al. [44], who divided the study sample into five age groups. In all groups, women were the majority declaring knowing how to cook. Van Der Horst et al. [3] observed that, in addition to men having lower cooking skills than women, a barrier in the preparation of homemade food, being overweight, and poor cooking skills were associated with the consumption of ready-to-eat meals. Thus, these variables are interconnected and all decrease diet quality, which can lead to overweight and obesity, especially in men [11,45].

Furthermore, our study found that eating out, such as eating in a restaurant, a cafeteria, an a la carte restaurant, and eating fast food, increased the chances of overweight and obesity. Frequently eating out compromises diet quality, increasing energy, total and saturated fat, sugar, and sodium intake and contributes to decreasing the consumption of fresh foods and micronutrients [46]. Strengthening this proposition, Tani et al. [2] found that, despite the benefits of homemade food, the consumption of these preparations declined in their residences when out-of-home food, such as fast food and convenience food, emerged. Kim et al. [47] observed a positive association between eating out and a high BMI among women. Therefore, eating out is related to a higher BMI [2,47].

Thus, it is essential to reinforce how the development of cooking skills is a significant modifying factor in encouraging people to cook. By strengthening these skills, diet quality can improve [2]. Some studies have shown an association between high cooking skills and the lower consumption of ready-to-eat meals and ultra-processed foods among adults [3,4,48]. Other studies have shown that interventions aimed at improving cooking skills increase culinary confidence and result in better food choices, such as the increased consumption of fruits and vegetables [48,49].

Effective culinary interventions were observed in the study by Bernardo et al. [8]: a randomized trial in which Brazilian university students participated in a six-week culinary intervention with weekly meetings, five practical cooking classes, and a food shopping selection workshop. The intervention successfully increased the access and availability of fruits and vegetables; culinary attitudes; self-efficacy for using culinary techniques; self-efficacy in using fruits, vegetables, and seasonings; and knowledge of cooking terms and techniques. In addition, Flego et al. [49], using the Jamie’s Ministry of Food Program as an intervention in Australian adults, improved culinary confidence and eating/cooking behaviors, contributing to a healthier diet for the participants. The intervention consisted of ten weeks with a 1.5 h weekly class. Like Bernardo et al. [8], the program emphasized cooking from scratch, nutritious meals, and good shopping choices. Thus, intervention programs should consider these aspects.

Interestingly, the bivariate analysis showed that there was a higher percentage of students with overweight or obesity, with both low and high culinary self-efficacy compared to students without overweight or obesity. This result shows that there seemed to be two profiles in the studied population: students with overweight or obesity who have high and low culinary self-efficacy. For students with overweight or obesity with low cooking self-efficacy, targeted actions to increase meal preparation and cooking skills could be interesting. For those with high culinary self-efficacy, actions aimed at preparing and consuming healthy foods in healthy preparations could be beneficial. However, in the multivariate analysis, culinary self-efficacy was not associated with overweight or obesity. Taking our results together (both the bivariate and multivariate analyses), the determinants of culinary self-efficacy could be further explored in other studies.

Culinary self-efficacy demonstrates confidence in cooking with respect to basic cooking skills. Our results suggest that for those with overweight and obesity confidence in cooking seems to decrease. Thus, developing culinary self-efficacy is important for healthy eating habits, such as a reduction in the consumption of processed foods and food autonomy, resulting in a better nutritional status [12,26,50].

We also found that students with overweight or obesity mainly had low-level knowledge of culinary techniques and terms. These students incorrectly answered questions involving roasting food, measuring cups, boiling water, and sautéing food. This technical knowledge and confidence in cooking are related to better diet quality and cooking using basic ingredients, which is an essential determinant of culinary behavior, according to Jomori et al. [1].

Thus, interventions in our population should address the creation of strategies to increase the frequency of cooking at home as a way of improving the quality of what is consumed, increasing the intake of fruits and vegetables, and reducing the consumption of ready-to-eat or convenience foods [8,51,52]. Studies point out that these culinary interventions present themselves as promising strategies to reduce the consumption of ready-to-eat and non-prepared foods at home [8,52].

Considering students’ food consumption and its possible relationship with changes in BMI, the COVID-19 pandemic was decisive for changes in people’s buying behavior and food consumption patterns [53]. This is because eating habits and behaviors can be affected by sociological, physiological, and psychological factors, and the pandemic period affected all of them [54], mainly due to social distancing to prevent the spread of the virus [24,50], which reduced trips to the supermarket and increased the use of delivery services [25] with a consequent decrease in vegetable intake and increased consumption of ultra-processed foods [26]. In addition, since the beginning of the pandemic, universities have closed their facilities and adopted distance learning, which has resulted in students dealing with an unexpected and potentially anxiety-producing situation [17,18]. As a result, there was an increase in food intake as anxiety can act as a trigger for people to use food in response to unfavorable emotions [19,20]. Thus, the increase in food intake associated with the higher consumption of industrialized foods may be related to the emergence of overweight in university students [20], which was observed in studies, such as those by Palmer et al. [55] and Tan et al. [53], who found weight gain in university students during confinement due to changes in eating patterns.

The present study has some limitations. The study involved students from a public university in Northeast Brazil, which was a particular group in terms of age, parental education, and ethnicity. Therefore, it is not possible to generalize the findings to other populations of undergraduate students from different Brazilian regions. Although we had a large sample size, sampling was not probabilistic, and there may have been selection bias, selecting those most interested in the topic under study. In the present study, the trend toward greater female involvement could be explained by their familiarity with the topic related to gender biases [12,50], as discussed. Nevertheless, several studies conducted online on other topics, such as food choice values, mental health, COVID-19, and fertility, showed greater female participation [41,56,57,58], which may reflect greater adherence of this group to this method of collecting data. Thus, these limitations do not invalidate the research objectives and the associations found.

For strengths, the study presented a large sample size, allowing a multivariate analysis, and cooking skills were characterized using a scale validated with Brazilian undergraduates [32]. Cooking skills were associated with overweight and obesity, conditions directly related to health. In Brazil, some documents make health promotion strategies viable, such as the Food and Nutrition Education Framework for Public Policies [59] and the Food Guide for the Brazilian Population [5]. However, no public policies promote healthy eating specifically for the university community or community-based culinary skill interventions. Thus, our results can contribute to developing public policies on food and nutrition education at the university. Promoting cooking skills in these students can contribute to healthy habits, diet quality, and better awareness of choosing and making better foods.

The present study showed that better self-efficacy in the use of fruits, vegetables, and seasonings, a culinary skill construct, was associated with less chances for overweight/obesity. Additionally, eating out of the home was associated with higher chances for overweight/obesity. Therefore, the development and encouragement of cooking skills should be part of health promotion activities. The university can be considered a privileged setting for the implementation of cooking courses that help raise awareness about food, culinary ingredients, preparing and eating meals that promote health and well-being, and for teaching students how to prepare foods in the most nutritious way. This can help the students involved apply the technical information in everyday practice, serving as an intervention for improving health via cooking skills.

## 5. Conclusions

Our study showed that better self-efficacy in using fruits, vegetables, and seasonings, which is a culinary skill construct, and sharing the responsibility for preparing meals were associated with fewer chances for overweight and obesity. Living with other people and eating out of the home were associated with higher chances for overweight and obesity. Considering the bivariate and multivariate analyses, our findings showed that overweight and obesity were associated with lower cooking skills, so the present study confirmed our hypothesis. Thus, the university environment emerges as a place to develop and encourage health promotion measures by facilitating the development of culinary skills.

## Figures and Tables

**Table 1:** Characterization of the studied undergraduates (*n* = 822).

Age (Years), Median (Q1–Q3)	23 (21.0–30.0)
Sex, *n* (%)	
Female	582 (70.8)
Male	240 (29.2)
Total	822 (100.0)
Ethnicity, *n* (%)	
White	426 (52.1)
Asiatic	07 (0.9)
Brown	308 (37.7)
Indigenous	02 (0.2)
Black	75 (9.2)
Total	822 (100.0)
Income, *n* (%)	
≤1.5 minimum wages	92 (12.9)
>1.5 minimum wages	621 (87.1)
Total	713 (100.0)
Body Mass Index, *n* (%)	
Underweight	63 (7.7)
Healthy weight	401 (48.8)
Overweight	238 (29.0)
Obesity	120 (14.6)
Total	822 (100.0)
Beneficiary of any aid from local university, *n* (%)	
Yes	253 (30.8)
No	569 (69.2)
Total	822 (100.0)
The student had children under 16 years, *n* (%)	
Yes	96 (11.7)
No	726 (88.3)
Total	822 (100.0)
Living arrangement, *n* (%)	
Alone	74 (9.0)
With parents or grandparents	503 (61.3)
With roommates	48 (5.8)
With partner or spouse	81 (9.9)
With children	16 (1.9)
With partner/spouse and children	77 (9.4)
Others	22 (2.7)
Total	822 (100.0)

**Table 2:** Studied undergraduates (*n* = 822) according to self-declaration of knowing how to cook, time available to cook per day, and overweight/obesity, according to sex.

Variables	Total *n* (%)	Sex		*p*-Value, Fisher’s Exact Test
		Male *n* (%)	Female *n* (%)	
Self-declaration of knowing how to cook				
Yes	695 (84.5)	189 (78.8)	506 (86.9)	0.004
No	127 (15.5)	51 (21.2)	76 (13.1)	
Total	822 (100)	240 (100)	582 (100)	
Time available for cooking/day				
≥2 h	449 (54.6)	149 (62.1)	300 (51.5)	0.007
<2 h	373 (45.4)	91 (37.9)	282 (48.5)	
Total	822 (100)	240 (100)	582 (100)	
With overweight or obesity				
Yes	358 (43.6)	116 (48.3)	242 (41.6)	0.089
No	464 (56.4)	124 (51.7)	340 (58.4)	
Total	822 (100)	240 (100)	582 (100)	

**Table 3:** Preparation and consumption of meals according to the presence of overweight/obesity in the studied undergraduates (*n* = 822).

Variables	Total *n* (%)	Overweight/Obesity		*p*-Value, Chi-Square or Fisher’s Exact Test
		Yes *n* (%)	No *n* (%)	
Self-reporting of knowing how to cook				
Yes	695 (84.5)	303 (84.5)	392 (84.5)	1.000
No	127 (15.5)	55 (15.4)	72 (15.5)	
Total	822 (100)	358 (100)	464 (100)	
How the student learned how to cook				
Family	659 (80.2)	286 (79.9)	373 (80.4)	0.860
Course	101 (12.3)	48 (13.4)	53 (11.4)	0.394
Internet	560 (68.1)	230 (64.2)	330 (71.1)	0.041
Books	201 (24.5)	92 (25.7)	109 (23.5)	0.513
Friends	163 (19.8)	66 (18.4)	97 (20.9)	0.427
TV	195 (23.7)	87 (24.3)	108 (23.2)	0.741
Alone	327 (39.8)	143 (39.9)	184 (39.7)	0.943
Place where the main meal is consumed				
Home	730 (88.8)	305 (85.2)	425 (91.6)	0.005
Out of home	82 (11.2)	53 (14.8)	39 (8.4)	
Total	822 (100)	358 (100)	464 (100)	
Place of meal away from home				
University restaurant	132 (16.1)	49 (13.7)	83 (17.9)	0.132
Restaurant per kilo	205 (24.9)	98 (27.4)	107 (23.1)	
A la carte restaurant	93 (11.3)	44 (12.3)	49 (10.6)	
Fast food	70 (8.5)	35 (9.8)	35 (7.5)	
Cafeteria	152 (18.5)	54 (15.1)	98 (21.1)	
Coffee shop	7 (0.9)	3 (0.8)	4 (0.9)	
Other places	163 (19.8)	75 (20.9)	88 (19.0)	
Total	822 (100)	358 (100)	464 (100)	
Responsibility for preparing the meal at home				
Only the student	175 (21.3)	95 (26.5)	80 (17.2)	0.031
The student and one other person	333 (40.5)	135 (37.7)	198 (42.7)	
The student and two other people	118 (14.4)	50 (14.0)	68 (14.7)	
Another person	148 (18.0)	59 (16.5)	89 (19.2)	
Two other people	48 (5.8)	19 (5.3)	29 (6.2)	
Total	822 (100)	358 (100)	464 (100)	
Other people who help preparing meals at home				
Mother	458 (70.8)	181 (68.8)	277 (72.1)	0.379
Father	81 (12.5)	24 (9.1)	57 (14.8)	0.039
Grandmother	50 (7.7)	15 (5.7)	35 (9.1)	0.134
Grandfather	2 (0.3)	1 (0.4)	1 (0.3)	1.000
Sister	90 (13.9)	39 (14.8)	51 (13.3)	0.644
Brother	22 (3.4)	7 (7.2)	15 (3.9)	0.509
Time available for cooking per day				
Greater than or equal to 2 h	373 (45.4)	158 (44.1)	215 (46.3)	0.572
Less than two hours	449 (54.6)	200 (55.9)	249 (53.7)	
Total	822 (100)	358 (100)	464 (100)	
Increased meal preparation during the pandemic				
Yes	698 (83.3)	300 (83.8)	389 (83.8)	1.000
No	133 (16.2)	58 (16.2)	75 (16.2)	
Total	822 (100)	358 (100)	464 (100)	
Living arrangement				
Alone	74 (9.0)	28 (7.8)	46 (9.9)	<0.0005
With parents or grandparents	503 (61.3)	193 (53.9)	310 (67.0)	
With roommates	48 (5.8)	19 (5.3)	29 (6.3)	
With partner/spouse	81 (9.9)	47 (13.1)	34 (7.3)	
With children	16 (1.9)	9 (2.5)	7 (1.5)	
With partner/spouse and children	77 (9.4)	52 (14.5)	25 (5.4)	
Others	22 (2.7)	10 (2.8)	12 (2.6)	
Total	821 (100)	358 (100)	463 (100)	

**Table 4:** Cooking skills according to the presence of overweight/obesity in the studied undergraduates (*n* = 822).

	Total *n* (%)	Overweight/Obesity		*p*-Value, Chi-Square
		Yes *n* (%)	No *n* (%)	
Availability of fruits and vegetables				
Low	82 (10.0)	42 (11.7)	40 (8.6)	0.261
Average	358 (43.6)	149 (41.6)	209 (45.0)	
High	382 (46.5)	167 (46.6)	215 (46.3)	
Total	822 (100)	358 (100)	464 (100)	
Culinary attitude				
Low	36 (4.4)	17 (4.7)	19 (4.1)	0.953
Average	336 (40.9)	149 (41.6)	187 (40.3)	
High	450 (54.7)	192 (53.6)	258 (55.6)	
Total	822 (100)	358 (100)	464 (100)	
Culinary behavior				
Low	57 (6.9)	31 (8.7)	26 (5.6)	0.352
Average	69 (8.4)	31 (8.7)	38 (8.2)	
High	696 (84.7)	296 (82.7)	400 (86.2)	
Total	822 (100)	358 (100)	464 (100)	
Culinary self-efficacy				
Low	37 (4.5)	21 (5.9)	16 (3.4)	0.026
Average	236 (28.7)	90 (25.1)	146 (31.5)	
High	549 (66.8)	247 (69.0)	302 (65.1)	
Total	822 (100)	358 (100)	464 (100)	
Self-efficacy in the consumption of fruits and vegetables				
Low	102 (12.4)	56 (15.6)	46 (9.9)	0.064
Average	196 (23.8)	88 (24.6)	108 (23.3)	
High	524 (63.7)	214 (59.8)	310 (66.8)	
Total	822 (100)	358 (100)	464 (100)	
Self-efficacy in the use of fruits, vegetables, and seasonings				
Low	24 (2.9)	16 (4.5)	8 (1.7)	0.042
Average	211 (25.7)	87 (24.3)	124 (26.7)	
High	587 (71.4)	255 (71.2)	332 (71.6)	
Total	822 (100)	358 (100)	464 (100)	
Knowledge of culinary terms and techniques				
Low	407 (49.5)	167 (46.6)	240 (51.7)	0.050
High	415 (50.5)	191 (53.4)	224 (48.3)	
Total	822 (100)	358 (100)	464 (100)	
Overall cooking skills				
Low	5 (0.6)	2 (0.6)	3 (0.6)	0.966
Average	277 (33.7)	122 (34.1)	155 (33.4)	
High	540 (65.7)	234 (65.4)	306 (65.9)	
Total	822 (100)	358 (100)	464 (100)	

**Table 5:** Logistic regression for variables associated with overweight and obesity in the studied undergraduates.

Independent Variables	OR (95% CI)	AOR (95% CI)
Sex		
Female	−	−
Male	1.31 (0.97–1.77)	1.39 (0.97–1.97)
Income		
>1.5 minimum wages	−	−
≤1.5 minimum wages	1.44 (0.92–2.23)	1.37 (0.86–2.182)
Living arrangement		
Alone	−	−
With parents or grandparents	1.02 (0.61–1.69)	2.06 (1.00–4.23)
With roommates	1.07 (0.51–2.26)	1.44 (0.61–3.37)
With partner/spouse	2.27 (1.19–4.32)	3.17 (1.42–7.04)
With children	2.11 (0.70–6.30)	1.72 (0.46–6.43)
With partner/spouse and children	3.41 (1.79–6.67)	5.10 (2.17–12.00)
Others	1.36 (0.52–3.58)	1.67 (0.54–5.17)
Responsibility for preparing the meal at home		
Only the student	−	−
The student and one other person	0.57 (0.39–0.83)	0.44 (0.26–0.74)
The student and two other people	0.61 (0.38–0.99)	0.54 (0.28–1.02)
Another person	0.55 (0.35–0.87)	0.42 (0.22–0.80)
Two other people	0.55 (0.28–1.05)	0.48 (0.20–1.12)
Place where the main meal is consumed		
Home	−	−
Out of home	1.89 (1.22–2.93)	1.89 (1.14–3.13)
Culinary self-efficacy		
Low	−	−
Average	0.47 (0.23–0.94)	0.56 (0.23–1.34)
High	0.62 (0.31–1.22)	0.68 (0.28–1.66)
Self-efficacy in the use of fruits, vegetables, and seasonings		
Low	−	−
Average	0.35 (0.14–0.85)	0.34 (0.11–1.00)
High	0.38 (0.16–0.91)	0.32 (0.11–0.95)

## Data Availability

Data described in the manuscript, code book, and analytic code will be made available upon request pending application and approval.

## References

[1] Jomori M.M., de Vasconcelos F.d.A.G., Bernardo G.L., Uggioni P.L., Proença R.P.d.C. (2018). The Concept of Cooking Skills: A Review with Contributions to the Scientific Debate. Rev. Nutr..

[2] Tani Y., Fujiwara T., Kondo K., Kondo K. (2020). Cooking Skills Related to Potential Benefits for Dietary Behaviors and Weight Status among Older Japanese Men and Women: A Cross-Sectional Study from the JAGES. Int. J. Behav. Nutr. Phys. Act..

[3] Van Der Horst K., Brunner T.A., Siegrist M. (2011). Ready-Meal Consumption: Associations with Weight Status and Cooking Skills. Public Health Nutr..

[4] Lam M.C.L., Adams J. (2017). Association between Home Food Preparation Skills and Behaviour, and Consumption of Ultra-Processed Foods: Cross-Sectional Analysis of the UK National Diet and Nutrition Survey (2008–2009). Int. J. Behav. Nutr. Phys. Act..

[5] Brazil, Ministry of Health (2014). Food Guide of the Brazilian Population.

[6] Fondevila-Gascón J.F., Berbel-Giménez G., Vidal-Portés E., Hurtado-Galarza K. (2022). Ultra-Processed Foods in University Students: Implementing Nutri-Score to Make Healthy Choices. Healthcare.

[7] Monteiro C.A., Cannon G., Moubarac J.C., Levy R.B., Louzada M.L.C., Jaime P.C. (2018). The Un Decade of Nutrition, the NOVA Food Classification and the Trouble with Ultra-Processing. Public Health Nutr..

[8] Bernardo G.L., Jomori M.M., Fernandes A.C., Colussi C.F., Condrasky M.D., da Costa Proenca R.P. (2018). Positive Impact of a Cooking Skills Intervention among Brazilian University Students: Six Months Follow-up of a Randomized Controlled Trial. Appetite.

[9] Munt A.E., Partridge S.R., Allman-Farinelli M. (2017). The Barriers and Enablers of Healthy Eating among Young Adults: A Missing Piece of the Obesity Puzzle: A Scoping Review. Obes. Rev..

[10] Bernardo G.L. (2017). Programa de Intervenção Sobre Habilidades Culinárias: Adaptação, Aplicação e Avaliação do Impacto Nas Práticas Alimentares de Estudantes Universitários no Brasil. Ph.D. Thesis.

[11] de Borba T.P., da Silva M.V., Jomori M.M., Bernardo G.L., Fernandes A.C., Proença R.P.d.C., Rockenbach G., Uggioni P.L. (2021). Self-Efficacy in Cooking and Consuming Fruits and Vegetables among Brazilian University Students: The Relationship with Sociodemographic Characteristics. Br. Food J..

[12] Wilson C.K., Matthews J.I., Seabrook J.A., Dworatzek P.D.N. (2017). Self-Reported Food Skills of University Students. Appetite.

[13] de Araujo T.A., de Medeiros L.A., Vasconcelos D.B., Dutra L.V. (2021). (In) Segurança Alimentar e Nutricional de Residentes Em Moradia Estudantil Durante a Pandemia Do COVID-19. Segur. Aliment. Nutr..

[14] Halpern B., Mancini M.C., de Melo M.E., Lamounier R.N., Moreira R.O., Carra M.K., Kyle T.K., Cercato C., Boguszewski C.L. (2022). Proposal of an Obesity Classification Based on Weight History: An Official Document by the Brazilian Society of Endocrinology and Metabolism (SBEM) and the Brazilian Society for the Study of Obesity and Metabolic Syndrome (ABESO). Arch. Endocrinol. Metab..

[15] Lin T.K., Teymourian Y., Tursini M.S. (2018). The Effect of Sugar and Processed Food Imports on the Prevalence of Overweight and Obesity in 172 Countries. J. Glob. Health.

[16] Sievert K., Lawrence M., Naika A., Baker P. (2019). Processed Foods and Nutrition Transition in the Pacific: Regional Trends, Patterns and Food System Drivers. Nutrients.

[17] Flaudias V., Iceta S., Zerhouni O., Rodgers R.F., Billieux J., Llorca P.M., Boudesseul J., Chazeron I.D.E., Romo L., Maurage P. (2020). COVID-19 Pandemic Lockdown and Problematic Eating Behaviors in a Student Population. J. Behav. Addict..

[18] Ramalho S.M., Trovisqueira A., de Lourdes M., Gonçalves S., Ribeiro I., Vaz A.R., Machado P.P.P., Conceição E. (2022). The Impact of COVID-19 Lockdown on Disordered Eating Behaviors: The Mediation Role of Psychological Distress. Eat Weight. Disord..

[19] Ammar A., Brach M., Trabelsi K., Chtourou H., Boukhris O., Masmoudi L., Bouaziz B., Bentlage E., How D., Ahmed M. (2020). Effects of COVID-19 Home Confinement on Eating Behaviour and Physical Activity: Results of the ECLB-COVID19 International Online Survey. Nutrients.

[20] Tavolacci M.P., Ladner J., Dechelotte P. (2021). COVID-19 Pandemic and Eating Disorders among University Students. Nutrients.

[21] Brazil M.E. ProUni. http://portal.mec.gov.br/ProUni.

[22] Brazil M.E. FIES. http://portal.mec.gov.br/fies-sp-1344319726.

[23] Brazil M.E. Pnaes. http://portal.mec.gov.br/pnaes.

[24] Yi-Chi C.Y., Wu P.L., Chiou W. (2021). Bin Thoughts of Social Distancing Experiences Affect Food Intake and Hypothetical Binge Eating: Implications for People in Home Quarantine during COVID-19. Soc. Sci. Med..

[25] Ben Hassen T., El Bilali H., Allahyari M.S., Berjan S., Fotina O. (2021). Food Purchase and Eating Behavior during the COVID-19 Pandemic: A Cross-Sectional Survey of Russian Adults. Appetite.

[26] Uggioni P.L., Elpo C.M.F., Geraldo A.P.G., Fernandes A.C., Mazzonetoo A.C., Bernardo G.L. (2020). Cooking Skills during the COVID-19 Pandemic. Rev. Nutr..

[27] Dezanetti T., Quinaud R.T., Caraher M., Jomori M.M. (2022). Meal Preparation and Consumption before and during the COVID-19 Pandemic: The Relationship with Cooking Skills of Brazilian University Students. Appetite.

[28] José M., Pinho S., Fernandes K.M., Rocha-Oliveira R. (2020). Educação, Tecnologias e COVID-19: O Que Nos Dizem Os Estudantes. Rev. Olhares.

[29] Jomori M.M. (2017). Adaptação transcultural e validação de um instrumento de identificação das Habilidades Culinárias e Alimentação Saudável em estudantes ingressantes de uma universidade brasileira. Ph.D. Thesis.

[30] WHO (1995). Physical Status: The Use and Interpretation of Anthropometry. Report of a WHO Expert Committee. World Health Organ. Tech. Rep. Ser..

[31] Jomori M.M., da Costa Proença R.P., Echevarria-Guanilo M.E., Bernardo G.L., Uggioni P.L., Fernandes A.C. (2017). Construct Validity of Brazilian Cooking Skills and Healthy Eating Questionnaire by the Known-Groups Method. Br. Food J..

[32] Jomori M.M., Quinaud R.T., Condrasky M.D., Caraher M. (2022). Brazilian Cooking Skills Questionnaire Evaluation of Using/Cooking and Consumption of Fruits and Vegetables. Nutrition.

[33] Jomori M.M., Caraher M., Bernardo G.L., Uggioni P.L., Echevarria-Guanilo M.E., Condrasky M., da Costa Proença R.P. (2021). How Was the Cooking Skills and Healthy Eating Evaluation Questionnaire Culturally Adapted to Brazil?. Cienc. Saude Colet..

[34] Lancaster K.G. (2009). Overweight and Obesity among Australian Youth: Associations with Family Background and Social Networks. Master’s Thesis.

[35] Djalalinia S., Yoosefi M., Shahin S., Ghasemi E., Rezaei N., Ahmadi N., Rezaei N., Azmin M., Rezaei S., Nasserinejad M. (2022). The Levels of BMI and Patterns of Obesity and Overweight during the COVID-19 Pandemic: Experience from the Iran STEPs 2021 Survey. Front. Endocrinol..

[36] Dinour L., Leung M.M., Tripicchio G., Khan S., Yeh M.C. (2012). The Association between Marital Transitions, Body Mass Index, and Weight: A Review of the Literature. J. Obes..

[37] Lemos A.H.D.C., Barbosa A.D.O., Monzato P.P. (2020). Women in Home Office during the COVID-19 Pandemic and the Work-Family Conflict Configurations. Rev. Adm. Empresas.

[38] Davis M.A., Murphy S.P., Neuhaus J.M., Gee L., Quiroga S.S. (2000). Community and International Nutrition Living Arrangements Affect Dietary Quality for U.S. Adults Aged 50 Years and Older: NHANES III 1988-1994 1. J. Nutr..

[39] Larson N.I., Perry C.L., Story M., Neumark-Sztainer D. (2006). Food Preparation by Young Adults Is Associated with Better Diet Quality. J. Am. Diet. Assoc..

[40] Ducrot P., Fassier P., Méjean C., Allès B., Hercberg S., Péneau S. (2016). Association between Motives for Dish Choices during Home Meal Preparation and Weight Status in the Nutrinet-Santé Study. Nutrients.

[41] Murakami K., Shinozaki N., Yuan X., Tajima R., Matsumoto M., Masayasu S., Sasaki S. (2022). Food Choice Values and Food Literacy in a Nationwide Sample of Japanese Adults: Associations with Sex, Age, and Body Mass Index. Nutrients.

[42] Taillie L.S. (2018). Who’s Cooking? Trends in US Home Food Preparation by Gender, Education, and Race/Ethnicity from 2003 to 2016. Nutr. J..

[43] Wolfson J.A., Ishikawa Y., Hosokawa C., Janisch K., Massa J., Eisenberg D.M. (2021). Gender Differences in Global Estimates of Cooking Frequency Prior to COVID-19. Appetite.

[44] García-González Á., Achón M., Alonso-Aperte E., Varela-Moreiras G. (2018). Identifying Factors Related to Food Agency: Cooking Habits in the Spanish Adult Population—A Cross-Sectional Study. Nutrients.

[45] Wien A., Alm S., Altintzoglou T. (2021). The Role of Identity and Gender in Seafood Cooking Skills. Br. Food J..

[46] Gesteiro E., García-Carro A., Aparicio-Ugarriza R., González-Gross M. (2022). Eating out of Home: Influence on Nutrition, Health, and Policies: A Scoping Review. Nutrients.

[47] Kim H.J., Oh S.Y., Choi D.W., Park E.C. (2019). The Association between Eating-out Rate and BMI in Korea. Int. J. Environ. Res. Public Health.

[48] Reicks M., Trofholz A.C., Stang J.S., Laska M.N. (2014). Impact of Cooking and Home Food Preparation Interventions Among Adults: Outcomes and Implications ForFuture Programs. J. Nutr. Educ. Behav..

[49] Flego A., Herbert J., Waters E., Gibbs L., Swinburn B., Reynolds J., Moodie M. (2014). Jamie’s Ministry of Food: Quasi-Experimental Evaluation of Immediate and Sustained Impacts of a Cooking Skills Program in Australia. PLoS ONE.

[50] Al-Domi H., AL-Dalaeen A., AL-Rosan S., Batarseh N., Nawaiseh H. (2021). Healthy Nutritional Behavior during COVID-19 Lockdown: A Cross-Sectional Study. Clin. Nutr. ESPEN.

[51] Smith L.P., Ng S.W., Popkin B.M. (2013). Trends in US Home Food Preparation and Consumption: Analysis of National Nutrition Surveys and Time Use Studies from 1965–1966 to 2007–2008. Nutr. J..

[52] Robson S.M., Stough C.O., Stark L.J. (2016). The Impact of a Pilot Cooking Intervention for Parent-Child Dyads on the Consumption of Foods Prepared Away from Home. Appetite.

[53] Tan S.T., Tan C.X., Tan S.S. (2022). Changes in Dietary Intake Patterns and Weight Status during the COVID-19 Lockdown: A Cross-Sectional Study Focusing on Young Adults in Malaysia. Nutrients.

[54] Yılmaz H.Ö., Aslan R., Unal C. (2020). Effect of the COVID-19 Pandemic on Eating Habits and Food Purchasing Behaviors of University Students. Kesmas.

[55] Palmer K., Bschaden A., Stroebele-Benschop N. (2021). Changes in Lifestyle, Diet, and Body Weight during the First COVID 19 ‘Lockdown’ in a Student Sample. Appetite.

[56] Chen T., Lucock M. (2022). The Mental Health of University Students during the COVID-19 Pandemic: An Online Survey in the UK. PLoS ONE.

[57] Ghazawy E.R., Ewis A.A., Mahfouz E.M., Khalil D.M., Arafa A., Mohammed Z., Mohammed E.F., Hassan E.E., Hamid S.A., Ewis S.A. (2020). Psychological Impacts of COVID-19 Pandemic on the University Students in Egypt. Health Promot. Int..

[58] Prior E., Lew R., Hammarberg K., Johnson L. (2019). Fertility Facts, Figures and Future Plans: An Online Survey of University Students. Hum. Fertil..

[59] Brazil M.S.D.F.H. (2012). Food and Nutrition Education Framework for Public Policies.

